# Respiratory viruses in mechanically ventilated patients: a pilot study

**DOI:** 10.1186/s12890-020-1082-5

**Published:** 2020-02-13

**Authors:** Raquel Nazareth, Maria-Jesus Chasqueira, Maria-Lúcia Rodrigues, Carolina Paulino, Catarina Conceição, Lia Lêdo, Úrsula Segura, Madalena Santos, António Messias, Pedro Póvoa, Paulo Paixão

**Affiliations:** 1 Hospital Beatriz Ângelo, Avenida Carlos Teixeira, 3, 2674-514 Loures, Portugal; 2 Centro de Estudos de Doenças Crónicas, CEDOC, Faculdade de Ciências Médicas|NOVA Medical School, Campo Mártires da Pátria, 130, 1169-056 Lisbon, Portugal; 3 Hospital São Francisco de Xavier, Estrada Forte do Alto Duque, 1449-005 Lisbon, Portugal; 4 Hospital Curry Cabral, Centro Hospitalar de Lisboa Central, Rua da Beneficiência n° 8, 1069-166 Lisbon, Portugal; 5 Center for Clinical Epidemiology and Research Unit of Clinical Epidemiology, OUH Odense University Hospital, Odense, Denmark; 6 Clinical Pathology Laboratory (Synlab), Hospital da Luz, Av Lusíada, 100, 1500-650 Lisbon, Portugal

**Keywords:** Human microbiome, Respiratory virome, Respiratory viruses, Real-time PCR

## Abstract

**Background:**

Respiratory virome is an integral part of the human microbiome and its characterization may contribute to a better understanding of the changes that arise in the disease and, consequently, influence the approach and treatment of patients with acute lower respiratory infections.

The aim of this study was to evaluate the presence of respiratory viruses in the lower airways of individuals undergoing invasive mechanical ventilation, with and without acute lower respiratory infection (respectively WRI and WORI groups).

**Methods:**

We studied 44 mini-bronchoalveolar lavage samples (collected with a double catheter, Combicath® kit) from patients with mean age in the seventh decade, 20 from WORI group and 24 from WRI group, who were hospitalized for acute respiratory failure in Intensive Care Units of two hospitals in the Lisbon area. Real-time PCR was applied to verify analyse the presence of 15 common respiratory viruses (adenovirus, human bocavirus, influenza virus A and B, repiratory syncytial virus, human parainfluenza virus types 1, 2, 3 and 4, human enterovirus, human rhinovirus, human metapneumovirus, human coronavirus group 1 (229E, NL63) and 2 (OC43, HKU1).

**Results:**

Respiratory viruses were detected in six of the 20 patients in the WORI group: influenza AH3 (*n* = 2), parainfluenza virus 1/3 (n = 2), human rhinovirus (n = 2), respiratory syncytial virus (*n* = 1) and human metapneumovirus (n = 1).

In the WRI group, respiratory viruses were detected in 12 of the 24 patients: influenza AH3 (*n* = 3), human rhinovirus (n = 3), respiratory syncytial virus (n = 3), human metapneumovirus (n = 3), human bocavirus (n = 2) and human enterovirus (n = 1). Simultaneous detection of two viruses was recorded in two samples in both groups.

**Conclusions:**

The results of this study suggest the presence of common respiratory viruses in the lower respiratory tract without causing symptomatic infection, even in carefully collected lower samples. This may have important implications on the interpretation of the results on the diagnostic setting.

## Background

The human microbiome was firstly defined by Joshua Lederberg in 2001, as a community of microorganisms that live in our organism, establishing a relationship of commensalism, symbiosis or pathogenicity [1].

Due to several factors, the study of the human virome has been neglected in comparison with the bacterial microbiome, namely the heterogeneity of the viral genome that leads to the absence of universal molecular markers, such as 16S ribosomal RNA for bacteria or fungal internal transcribed spacer region for fungi, the difficulty in working with small biomass samples, the interference of genetic material from the host, inadequate bioinformatic instruments for its analysis and the absence of robust databases [2, 3]. In the particular case of lung virome, there is also the problem of the sample collection, unlike with other mucosal surfaces which are easily accessible.

Until recently, the lung was considered a sterile organ [4], which was explained by the protective mechanisms of the upper airways and the barrier function of the mucosa of the lower airways. The presence of bacteria in the lower airways was interpreted as a pathological phenomenon, based on cultural microbiological information [5]. It was explained as transient migration by microaspiration, reflecting the composition of the upper respiratory tract, although the biomass would be lower. It was further conceded that the lower respiratory tract microbiome could be related to contamination of the upper tract during sampling [6]. According to Dickson and Huffnagle, the notion that the lungs are sterile is still frequently stated in textbooks, virtually always without citation [7]. Respiratory culture-based protocols sought only to identify clinically significant pathogens [8].

At the beginning of the current decade, studies based on culture-independent methods have shown the presence of a small amount of bacterial communities in the lungs of healthy, non-smoker individuals, with some diversity in their elements. There are few studies available in the literature on the characterization of the respiratory tract virome, especially of the lower airways [9–14]. Essentialy, most of the studied population was in the pediatric age and the majority of them had respiratory infection. Most of the observations made on these studies were based on upper respiratory tract samples, like nasopharyngeal swabs and nasal mucosa exudates, because of its greater accessibility, assuming that the results obtained could translate what was happening in the lower airways.

The objective of this study was to evaluate the presence of respiratory viruses in the lower airways of individuals with and without acute lower respiratory infection undergoing invasive mechanical ventilation. In order to achieve this, we evaluated the presence of the most common respiratory viruses in the population [15], in lower airway samples collected from individuals with and without respiratory infection, undergoing mechanical ventilation, using real-time Polymerase Chain Reaction and Reverse Transcription-Polymerase Chain Reaction (respectively PCR and RT-PCR) techniques.

## Methods

### Study design

This was a prospective observational study analyzing mini-bronchoalveolar lavage (mini-BAL) samples of invasive mechanical ventilated patients from two polivalent Intensive Care Units (ICUs) of Lisbon district, in Portugal. Patients were divided in two groups; WORI group included patients admitted for causes other than respiratory infection and not receiving antibiotic therapy (respiratory symptoms were excluded at time of admission, namely cough and sputum; chest x-ray was performed in all the patients to exclude lower respiratory infection. It was not possible to obtain reliable information about the presence of respiratory symptoms before admission nor about influenza vaccination history). WRI group included patients admitted for acute respiratory infection and on antibiotic therapy. All patients enrolled in the study required endotracheal intubation to treat their acute respiratory failure.

Exclusion criteria were age less than 18 years, pregnancy, immunosupression and antiviral therapy on admission. The participants, or their legal representatives, were informed about the study objectives and their signed consents were obtained previously to the sample collection. The Ethic Committees of both participating centers approved the study protocol.

Patient demographics, comorbidities, Acute Physiology and Chronic Health Evaluation (APACHE) II score and Simplified Acute Physiology Score (SAPS) II, admission diagnosis and ICU clinical outcome were recorded.

Sample collection was performed with a Combicath® kit, between December 2016 and March 2017.

### Mini-BAL procedure

A protected mini-BAL (with a double catheter, Combicath® kit (Plastimed, Saint-Leu-La Forêt, France)) was performed on the first 24 h after the tracheal intubation and invasive mechanical ventilation. A combicath was introduced blindly through the oro-tracheal tubeuntil its end on the lower third of the trachea and wedged in the bronchial tree. Mini-BAL samples were obtained by intilling 2 mL of room temperature saline solution (0.9%), followed by gentle suction after the infusion of each aliquot. Samples were stored at − 80 °C until processed.

### Laboratory methods

Clinical samples were processed using the QIAmp MinElute Virus Spin kit for extraction, according to the manufacturer’s instructions (Qiagen, Valencia, CA).

“In house” real-time Taqman PCR and RT-PCR techniques, detailed elsewhere [16], were used for the detection of DNA and RNA respiratory viruses: adenovirus, human bocavirus (HBoV), influenza virus A/B, respiratory syncytial virus (RSV), human parainfluenza virus (HPIV) types 1/3 and 2/4, human enterovirus (HEV), human rhinovirus (HRV), human metapneumovirus (HMPV), human coronavirus (HCoV) group 1 (229E, NL63) and 2 (OC43, HKU1).

### Data analysis

Descriptive analysis was performed, values are expressed as percentages for discrete variables, or as the mean for continuous variables.

## Results

### Patient decription

A total of 44 patients were included in this study, 20 from the WORI group, and 24 from the WRI group. Baseline patient characteristics and admission diagnoses are summarized in Table 1. The median age was 68.3 years (20–87 years) and 56.3% of the patients were male. APACHE II and SAPS II medium scores were 23.5 (3–49) and 53.4 (12–103), respectively. The medium ICU length of stay was 9.7 days (1–25 days). A total of 17 patients died in the ICU.

**Table 1:** Demographics, comorbidities and ICU clinical outcome of the study subjects: WORI group and WRI group

	Total (*n* = 44)	WORI group (*n* = 20)	WRI group (*n* = 24)
Age (years)	68.3 (20–87)	65.3 (20–87)	70.7 (42–87)
Male, *n* (%)	25 (56.8)	12 (60)	13 (54.2)
APACHE II	23.5 (3–49)	22.9 (13–37)	23.9 (3–49)
SAPS II	53.4 (12–103)	56.3 (22–103)	51 (12–93)
ICU length of stay (days)	9.7 (1–25)	9.9 (1–25)	9.6 (3–25)
ICU mortality, n (%)	17 (38.6)	10 (50)	7 (29.2)
Admission diagnosis, n			
Respiratory	11	0	11
Cardiovascular	3	1	2
Shock	17	13	4
Cardio-respiratory arrest	5	1	4
Neurologic	7	4	3
Metabolic	1	1	0

APACHE II Acute Physiology and Chronic Health Evaluation score (0–71); SAPS II, Simplified Acute Physiology Score (0–163);

*ICU* intensive care unit, *WRI* with respiratory infection, *WORI* without respiratory infection

### Viruses detected using real-time PCR

The viruses detected are depicted on Table 2. In the WORI group, 6 of the 20 patients were positive and in the WRI group 12 of the 24 patients were positive. The Cycle Threshold (Ct) varied between 16.14 and 28.78 on WORI group and between 14.52 and 36.86, on WRI group.

**Table 2:** Respiratory virus detected by RT-PCR in the two groups

	WORI group (*n* = 20)	WRI group (n = 24)
N° positive samples	6	12
> 1 virus	2	3
Influenza A(H3)	2	3
RSV	1	3
HRV	2	3
HMPV	1	3
HPIV 1/3	2	0
HEV	0	1
HBoV	0	2

In WORI group, the following viruses were identified: Influenza AH3, HPIV 1/3, RSV, HMPV and HRV. In two samples two viruses were detected simultaneously, Influenza AH3 and HRV in one sample and HMPV and HRV in the other one. Both patients died in the ICU. Influenza viruses AH3, HPIV 1/3 and HRV were present in more than one sample.

In the WRI group were detected: Influenza AH3, RSV, HMPV, HRV, HEV and HBoV. Influenza virus AH3, RSV, HMPV and HRV were each identified in three samples. HBoV was identified in two samples and HEV in only one sample. There were three cases of association of two viruses, HEV and HRV, Influenza AH3 with HMPV and HMPV with HBoV. There were six cases of bacterial co-infection with *Staphylococcus aureus* (*N* = 2), *Moraxella catarrhalis* (N = 2) and *Streptococcus pneumoniae* (N = 2), detected by the routine microbiological procedure in cases of lower respiratory infection. None of these nine patients with viral or bacterial and viral co-infection died in the ICU.

In WORI group, patients with positive samples (WORI+) were older than patients with negative samples (WORI-) (74.2 vs. 61.6 years). Also, WORI+ patients had higher mean severity scores (APACHE II 25.3 vs. 21.9, SAPS II 59.8 vs. 54.8), lower ICU length of stay (4.8 vs. 12.1 days) and invasive mechanical ventilation days (2.3 vs. 9.5) and higher mortality rate (66.7% vs. 42.9%), (Table 3).

**Table 3:** Patients characteristics in each subgroup of results (average values)

	WORI group (n = 20)		WRI group (n = 24)	
	Virus positive (WORI+) (*n* = 6)	Virus negative (WORI-) (*n* = 14)	Virus positive (WRI+) (*n* = 12)	Virus negative (WRI-) (*n* = 12)
Age (years)	74.2	61.6	70.7	70.7
APACHE II	25.3	21.9	20.5	27.4
SAPS II	59.8	54.8	45	56.9
ICU length of stay (days)	4.8	12.1	9.1	10
Mechanical ventilation (days)	2.3	9.5	5.2	5.1
ICU mortality, *n* (%)	4 (66.7)	6 (42.9)	2 (16.7)	5 (41.7)

Concerning to WRI group, the mean age was the same (70.7 years) in both subgroups of patients, WRI+ and WRI- (Table 3). The severity scores presented higher values in the subgroup WRI- (APACHE II 27.4 vs. 20.5, SAPS II 56.9 vs. 45). ICU length of stay and invasive mechanical ventilation days were similar in both subgroups (9.1 vs. 10 and 5.2 vs. 5.1, respectively). ICU mortality was lower in the WRI+ subgroup (16.7% vs. 41.7%).

The subgroup WORI+ had higher median age (74.2 vs. 70.7 years) and higher values of severity scores (APACHE II 25.3 vs. 20.5; SAPS II 59.8 vs. 45) than WRI+ but lower ICU length of stay (4.8 vs. 9.1) and mechanical ventilation days (2.3 vs. 5.2). ICU mortality was higher in the WORI+ subgroup than in the WRI+ subgroup (66.7% vs. 16.7%).

## Discussion

In our study it was possible to identify common respiratory viruses in samples of the lower respiratory tract of adults under invasive mechanical ventilation, regardless of the presence or absence of acute lower respiratory infection, through real-time PCR techniques. In the field of pulmonary virology investigation, several factors make this study innovative: the age and type of patients, the inclusion of two groups of patients, with (WRI group) and without acute lower respiratory infection (WORI group), and also the type of samples (protected samples from the distal lower respiratory tract). According to Luyt et al, BAL is the respiratory sample of choice to evaluate lung infection [17].

Nucleic acid extraction was performed with the same commercial kit used in the study of Wang et al [13], who analized the respiratory virome of healthy and severe acute respiratory infection children through metagenomic analysis. We used “in-house” real-time PCR and RT-PCR techniques that were tested and validated long before [16].

Thirty percent of the patients belonging to WORI group had common respiratory viruses in the distal lower respiratory tract. According to data from the pediatric population [13], lung virome of asymptomatic individuals is less diverse than that of infected individuals, and consists mostly, of members of the *Anelloviridae* family, with a lower percentage of common epidemic respiratory viruses. In the study of Wang et al [13], samples from infected patients had six to seven-fold more viral pathogens than the WRI group. These authors suggested that the viral infection may be asymptomatic and, occasionally prolonged, making controversial the interpretation of a positive PCR for some viruses.

. In a study conducted by Choi et al [18], the main respiratory viruses associated with severe pneumonia in adults admitted in ICU were HRV (23.6%), HPIV (20.8%), HMPV (18.1%), influenza (16.7%) and RSV (13.9%), according to data obtained from real-time PCR analysis in bronchoalveolar lavage samples. In the study by Xu et al [14], 368 samples collected with nasopharyngeal swab from infected children were analyzed with real-time PCR**,** using a panel of 18 respiratory viruses. The percentage of positive samples was 58.97%.

In our study, half of the WRI group patients had positive samples. Comparing the two groups, influenza AH3 was the most prevalent virus, coinciding with the peak of influenza in Portugal. RSV, HMPV and HRV were common to both groups. Differences were found with HPIV 1/3, which was more prevalent in uninfected patients, and HEV and HBoV that were**,** only found in WRI group. The analysis of the Ct values showed similar viral loads in both groups, although with a slightly tendency for lower Ct values in WORI group. The small number of samples did not allow us to draw conclusions about possible differences in viral loads between infected and uninfected individuals.

It may be hypothesized that some respiratory viruses, such as influenza, RSV, HMPV and HRV, may transiently colonize the mucosa of the tracheobronchial tract at times of increased viral activity, whereas others, such as HPIV 1/3, might be prolonged colonizers. However, only a longitudinal study could determine the extent of the colonization period and thus solve this important issue. In addition, we need to know the meaning of its presence, if they are only bystanders, even during an acute respiratory infection, or if they are responsible for symptomatic infections of the lower respiratory tract.

Our study has some limitations, including a small sample size and a limited number of centers involved, which does not allow us to make inferences about mortality between the groups and subgroups. In addition, all the patients in both groups were severely ill. Therefore, the observations made in this study may not be generalizable to other groups, in particular to healthy populations.

Although signs and symptoms of respiratory infection were excluded at admission in the WORI group, viral respiratory infections in the last weeks preceding hospitalization cannot be excluded, and therefore viral detection in some of the cases could be the result of a recent infection and not an extended stay in the lower respiratory tract.

In this study a protected mini-BAL (with a double catheter, Combicath® kit) was used in order to reduce the upper level contamination. However, it is possible that contamination from upper respiratory secretions, mainly due to the ventilation process, may have had an important contribution to the detection rate observed in this study.

Another limitation relates with the methodology used: although real-time PCR is currently the gold-standard for the diagnosis of viral infections [19], this methodology is limited to specific target sequences. In fact, only the most common respiratory viruses were investigated. According to Willner et al [9], PCR-based studies confer an incomplete airway virome picture and little opportunity for the discovery of new agents, as compared to metagenomics, a technique independent of genomic sequences. In addition, viruses like herpesvirus simplex, cytomegalovirus or torque teno virus can be present in the distal airway mucosa [13, 20–22], and therefore may have been missed with our PCR strategy. However, despite this limitation, the main respiratory viruses were properly searched, and this is the group of major concern, when dealing with respiratory infections. Another important advantage of this study was the focus on a population undergoing invasive mechanical ventilation, allowing the collection of samples from the distal airways, although blindly, which is usually a limitation in this type of studies.

## Conclusions

The results of this study suggest the presence of common respiratory viruses in the lower respiratory tract without determining symptomatic infection even in carefully collected lower samples. This may have important implications on the interpretation of the results on the diagnostic setting.

The accomplishment of a longitudinal study would allow to elucidate the length of stay of these viruses in the respiratory tract, and the existence or not of a permanent viral community in this environment.

## Abbreviations

APACHE II: Acute Physiology and Chronic Health Evaluation score
BAL: Bronchoalveolar lavage
Ct: Cycle Threshold
DNA: Deoxyribonucleic acid
HBoV: Human bocavirus
HCoV: Human coronavirus
HEV: Human enterovirus
HMPV: Human metapneumovirus
HPIV: Human parainfluenza virus
HRV: Human rhinovirus
ICU: Intensive Care Unit
PCR: Polymerase chain reaction
RNA: Ribonucleic acid
RSV: Respiratory syncytial virus
RT-PCR: Reverse Transcription-Polymerase Chain Reaction
SAPS II: Simplified Acute Physiology Score
WORI-: Patients in study group with negative samples
WORI: Study group
WORI: Without respiratory infection
WORI+: Patients in study group with positive samples
WRI: Control group
WRI-: Patients in control group with negative samples
WRI: With respiratory infection
WRI+: Patients in control group with positive samples.
